# Pulsed Field Energy in Atrial Fibrillation Ablation: From Physical Principles to Clinical Applications

**DOI:** 10.3390/jcm13102980

**Published:** 2024-05-18

**Authors:** Nicola Pierucci, Marco Valerio Mariani, Domenico Laviola, Giacomo Silvetti, Pietro Cipollone, Antonio Vernile, Sara Trivigno, Vincenzo Mirco La Fazia, Agostino Piro, Fabio Miraldi, Carmine Dario Vizza, Carlo Lavalle

**Affiliations:** 1 Department of Cardiovascular, Respiratory, Nephrological, Aenesthesiological and Geriatric Sciences “Sapienza”, University of Rome, 00161 Rome, Italy; npierucci@gmail.com (N.P.); marcoval.mariani@gmail.com (M.V.M.); domenico.laviola@uniroma1.it (D.L.); giacomo.silvetti@uniroma1.it (G.S.); pietro.cipollone@uniroma1.it (P.C.); antonio.vernile@uniroma1.it (A.V.); sara.trivigno3@gmail.com (S.T.); agostino.piro@uniroma1.it (A.P.); fabio.miraldi@uniroma1.it (F.M.); dario.vizza@uniroma1.it (C.D.V.); 2 Texas Cardiac Arrhythmia Institute, St. David’s Medical Center, Austin, TX 78705, USA; vmirco.lafazia@gmail.com

**Keywords:** atrial fibrillation, pulse field ablation, catheter ablation, arrhythmia, new technologies

## Abstract

Atrial fibrillation, representing the most prevalent sustained cardiac arrhythmia, significantly impacts stroke risk and cardiovascular mortality. Historically managed with antiarrhythmic drugs with limited efficacy, and more recently, catheter ablation, the interventional approach field is still evolving with technological advances. This review highlights pulsed field ablation (PFA), a revolutionary technique gaining prominence in interventional electrophysiology because of its efficacy and safety. PFA employs non-thermal electric fields to create irreversible electroporation, disrupting cell membranes selectively within myocardial tissue, thus preventing the non-selective damage associated with traditional thermal ablation methods like radiofrequency or cryoablation. Clinical studies have consistently shown PFA’s ability to achieve pulmonary vein isolation—a cornerstone of AF treatment—rapidly and with minimal complications. Notably, PFA reduces procedure times and has shown a lower incidence of esophageal and phrenic nerve damage, two common concerns with thermal techniques. Emerging from oncological applications, the principles of electroporation provide a unique tissue-selective ablation method that minimizes collateral damage. This review synthesizes findings from foundational animal studies through to recent clinical trials, such as the MANIFEST-PF and ADVENT trials, demonstrating PFA’s effectiveness and safety. Future perspectives point towards expanding indications and refinement of techniques that promise to improve AF management outcomes further. PFA represents a paradigm shift in AF ablation, offering a safer, faster, and equally effective alternative to conventional methods. This synthesis of its development and clinical application outlines its potential to become the new standard in AF treatment protocols.

## 1. Introduction

Atrial fibrillation (AF) is the most common sustained cardiac arrhythmia affecting 1–2% of the general population, with a worldwide prevalence that has increased 3-fold over the last 50 years [1,2].

AF patients have an increased risk of stroke and heart failure, hospitalization, and cardiovascular death with a significant reduction in functional capacity and quality of life [3,4].

Because of these important consequences, early recognition and therapy of AF are of pivotal importance. The pathogenesis of AF is complex, and several studies reported that pulmonary vein (PV) foci play an important role in both initiation and perpetuation of this arrhythmia [5,6,7]. 

The primary goals of treating patients with AF include improving symptoms, managing heart rate or rhythm, and minimizing the risk of stroke [8]. Furthermore, we now know from numerous studies that the ablation procedure is important in reducing the need for rehospitalizations and also reducing death in patients with heart failure and AF (CASTLE-AF trial) [9], as well as the reduction of mortality in patients with end-stage heart failure and AF (CASTLE-HTx trial) [10].

Classic treatment with antiarrhythmic drugs (AADs) may modulate AF burden but usually does not lead to a consistent effect and may result in treatment failure. Over the past years catheter ablation has emerged as an important treatment strategy for symptom improvement and rhythm control [11].

The European guidelines of AF recommend AF catheter ablation in symptomatic patients refractory to drugs or with tachycardiomyopathy [8,11,12] to improve symptoms of AF recurrences in patients with paroxysmal AF, persistent AF without major risk factors for AF recurrence (Class IA), or persistent AF with major risk factors for AF recurrence (Class IB). AF catheter ablation for PVI should be considered for rhythm control after failure or intolerance to beta-blocker treatment to improve symptoms of AF recurrences in patients with paroxysmal and persistent AF (Class IIB) [8]. However, the indications are continuously expanding, especially in light of promising results regarding safety and efficacy obtained in trials such as the EAST-AFNET 4 trial [13] that demonstrated that an early rhythm control strategy to maintain sinus rhythm led to a lower risk of cardiovascular events such as death from cardiovascular causes, stroke, or hospitalization related to heart failure or acute coronary syndrome when compared to usual care, which primarily focused on rate control and anticoagulation. Moreover, trials have studied indications to ablation in more specific patient populations, such as those affected by immunodeficiency [14] or cardiotoxicity [15].

In general, rhythm control strategies exhibit greater evidence compared to rate control in the management of atrial fibrillation. Acutely, rhythm control can be achieved through electrical and pharmacological cardioversion. It is important to consider that although the rate of spontaneous cardioversion is high in certain types of patients with symptomatic AF, especially those with the absence of heart failure, small atrial size, recent-onset AF, rapid atrial fibrillatory rate, and the relationship between a previous AF episode and heart rate/blood pressure [16], as predicted by certain scoring systems, including a recent one published by Mariani et al. [17,18], there are other categories of patients in the chronic setting, particularly those with more severe cardiac conditions, who benefit much more from early interventional treatment than the use of AADs [18] as evidenced by the ATTEST trial [19]. 

The earliest demonstration of an AF trigger from within the PV by Haïssaguerre and colleagues in 1998 was a milestone in the field of cardiac electrophysiology, revealing a novel mechanistic pathway underlying AF development as well as a new potential target for treatment [5].

Catheter-based ablation has established itself as a mainstay in the rhythm control strategy of AF, and it has become the most common ablation procedure performed in electrophysiology worldwide [20,21]. PV isolation (PVI) remains the cornerstone of all ablation procedures irrespective of patient characteristics and AF pattern. Many ablation techniques have been developed over the years and different studies have demonstrated the value that each of them has in the treatment of AF. 

The two most common ablation energy sources are radiofrequency (RF) energy and cryoenergy. Three major prospective randomized controlled trials, FreezeAF [22], FIRE AND ICE [23], and CIRCA-DOSE [24], have assessed the comparability of cryoballoon ablation (CB) and RF ablation in patients with paroxysmal atrial fibrillation (PAF). The findings from these trials indicate that both approaches exhibit similar levels of efficacy and safety.

In addition to RF and cryoenergy, lasers represent another energy source feasible for AF ablation. Three generations of laser balloon ablation systems have been developed (namely HeartLight, HeartLight Excalibur, and HeartLight X3 by CardioFocus, Marlborough, MA, USA) and several studies have demonstrated the non-inferiority of lasers compared to RF or cryoablation systems in terms of safety and efficacy, with a success rate ranging from 61.7% to 82.3% [25,26,27,28].

Over the last two decades, there has been significant evolution in AF ablation procedures, with many different ablation tools to enhance safety and effectiveness [29], such as contact force catheters [30] and high-power short-duration [31] and very-high-power short-duration [32] ablation protocols.

Pulsed field ablation (PFA) is currently the groundbreaking ablative approach that is already changing the panorama in interventional electrophysiology. It employs a non-thermal ablative mechanism in which cell death is obtained by applying ultra-short electrical pulses to induce pores in cell membranes [33,34]. Its main benefits are the selectivity for myocardial tissue and the notable reduction in ablation procedure duration [30,31].

In this review we will focus our attention on electroporation/PFA, describing the operating mechanism, the evidence in favor of this new technique, the limits, and future prospects.

## 2. Mechanisms of Pulsed Field Ablation

PFA is a novel ablation modality that involves the application of ultra-rapid (microseconds to nanoseconds) electrical pulses to generate strong electrical fields causing, among other effects, irreversible nano-scale pore formation in the cellular membrane (a phenomenon called “electroporation”) and, ultimately, cellular death [35]. 

The phenomenon of electroporation has been known for some time and is exploited in various fields, especially in oncology. In the treatment of cutaneous and subcutaneous cancer, as well as for the treatment of deep-seated tumors, this approach is known as electrochemotherapy (ECT) and utilizes reversible electroporation to introduce molecules such as bleomycin, cisplatin, and calcium into tumor cells with a cytotoxic effect [36].

In the field of gene electrotherapy, electroporation has led to the successful transfer of non-viral genes for gene therapies, vaccination, or immunotherapies [37]. When electroporation is used to ablate tumors, such as prostate tumors, pancreatic cancers, or liver tumors, about one hundred pulses are used to permanently alter the cell membrane. This leads to cell death induced by so-called irreversible electroporation (IRE). The pulses used are in the kV/cm range, also referred to as high-intensity electric pulses [38,39,40].

Pulsed electric field (PEF) systems use a direct current power supply to charge capacitors, which then discharge through a high-voltage switch. These discharges produce high voltages between electrodes, creating an electric field. The pulses typically range from nanoseconds to milliseconds, with voltages spanning hundreds of volts to tens of kilovolts. Pulse shapes can vary (square, rectangular, exponential decay), and the electric field can be monopolar, bipolar, or potentially omnipolar. The most common pulse shape used is the waveform [41]. Longer pulses (over 1 ms) are generated by capacitor discharge, square wave, or analog generators. Shorter pulses (usually in the nanosecond range) use a Blumlein generator (a type of pulsed power generator used to produce high-power pulses invented by Alan Dower Blumlein, a British engineer, in the 1930s) or resonant charging generators.

Classical electroporation protocols generate muscle contractions and are associated with pain during and after electrical stimulation [42]. Muscle contractions may also cause electrodes displacement during the treatment and thus jeopardize the treatment’s outcome. New protocols have been established which limit or avoid pain sensation and discomfort. Among these new protocols, some use microsecond electrical pulses, applied at high pulse repetition rate (high PRR), also called “high frequency” electroporation protocols. They can be used to deliver chemotherapeutics or to ablate tissues. In ablation procedures, the term “high-frequency irreversible electroporation” (H-FIRE) is often used [43].

When a cell membrane, which is mostly a non-conductive structure, encounters an electric field, charges accumulate on opposite sides of the cell membrane and lead to an induced transmembrane voltage (TMV) which is additional to the resting TMV. This induced TMV is present only while the electric field is applied. If the total TMV (induced plus resting) reaches a critical threshold, the cell membrane undergoes permeabilization, a process known as electroporation. The threshold for permeabilization varies according to the cell and is reported to be between 200 and 500 mV [44]. Moreover, the induced TMV in a spherical cell is determined by the steady-state Schwan equation in which the induced TMV is directly linked to both the applied electric field and the cell’s radius [35]. Larger cells exhibit a higher TMV, thus potentially experiencing electroporation at lower field intensities compared to smaller cells. Consequently, the cell radius emerges as a pivotal determinant influencing tissue selectivity in these processes. The effect is greater when the field is perpendicular to the membrane (h = 0 and h = 180). While these equations are important for understanding the basic principles and the effect of electric fields on a single cell, there are limitations to their application. First, they can only be applied to spherical cells and cardiac cells are not spherical. With a change in cell shape, further adjustments to the above equations are required [44]. 

Beyond theoretical and analytical calculations, there are additional tools available to calculate the induced TMV. One particularly useful and reproducible method involves the use of potentiometric dyes. These dyes can be integrated into the cell membrane, and their fluorescent properties are contingent upon the TMV. This proves beneficial when dealing with complex or irregular cell shapes. Among the widely employed fast potentiometric dyes is di-8-butylamino-naphthyl-ethylene-pyridinium-pro-pyl-sulfonate (di-8-ANEPPS). In a normal solution, this dye is non-fluorescent, but it becomes strongly fluorescent when bound to the lipid bilayer of the cell membrane [45]. 

Electroporation can either be transient, leading to viable cells after exposure to an electric field (reversible electroporation), or result in cell death if the exposure is prolonged (irreversible electroporation). Electroporation generally occurs in five steps: initiation, expansion, stabilization, resealing, and memory, each at different time points. The mechanism of cell death by electroporation occurs via an influx of Ca^2+^ ions into the cell from the extracellular space, disrupting calcium homeostasis significantly. This disturbance prompts the cell to expend energy reserves, specifically ATP, by activating Ca-ATPases and inhibiting ATP production in the mitochondria. Additionally, this process generates reactive oxygen species, further destabilizing the membrane and leading to cell death. The mechanism of cell death varies based on the parameters of the electric field applied. Cell death induced by nanosecond pulse duration appears to primarily result from apoptosis due to increased release of intracellular calcium. However, there is also growing evidence supporting necroptotic cell death in this context [46].

Although DC is the most common energy source, preliminary studies are exploring magnetic fields [47]. According to Faraday’s principles, a changing magnetic field induces an electric field, potentially offering an electrode-less and contact-less option. However, this method is in its early stages and mainly explored in non-cardiac studies presently. Pulse electric fields will also result in Joule heating and a temperature rise, but this is usually biological insignificant.

## 3. Animal Studies on Pulsed Field Ablation

The application of PFA in clinical practice has found a rational foundation in several laboratory studies, both on animal and human cells.

In the study of Ye et al. [48] two ablation modes, one for single-cell systems using an electrode cup and another for monolayer cell systems using inserts with electrode tips, were used to perform PFA on two types of line of embryonic rat cells, the myocardial cell H9C2 and the smooth muscle cell of thoracic aorta A7r5. In the monolayer cell system, H9C2 cells showed higher sensitivity to PFA compared to A7r5 cells, resulting in the shrinkage of the entire monolayer. The bidirectional pulse’s ablation effect was weaker than that of the two monopolar pulses. 

In the same study three Bama minipigs were used to verify the in vivo ablation effect of PFA, showing that bidirectional PFA effectively blocked electrical activity from the PV to the atrium with minimal muscle contraction, avoiding PV stenosis, which is a well-known adverse effect in RF ablation. This bidirectional PFA method proved to obtain a stable ablation of myocardial cells, maintained cell-to-cell connections, and reduced muscle contraction [48].

In another study, Avazzadeh et al. [49] aimed to explore the application of irreversible electroporation (IRE) for treating AF, focusing on identifying the ideal voltage thresholds for selective cell ablation within the cardiovascular system. Monophasic pulses were administered to various cell types (three different neuronal lines of pheochromocytoma cells derived from Rattus norvegicus adrenal glands, a somatic cell hybrid of rat embryonic dorsal root ganglion, mouse neuroblastoma, and human neuroblastoma cells, cardiomyocytes, and cardiac fibroblasts were assessed under different voltage conditions) in a cuvette suspension model. They evaluated cell viability under different voltage conditions. The findings revealed that neuronal lines and atrial cardiomyocytes exhibited less than 20% cell viability at an electric field of 1000 V/cm with a minimum of 30 pulses, showing no significant difference between them. However, cardiac fibroblasts had an optimal threshold at 1250 V/cm with a minimum of 50 pulses to induce cell death effectively, resulting in a higher death threshold than cardiomyocytes. The study observed immediate or delayed cell death, with some cell membranes resealing after three hours, but no substantial difference was noted between treatments after 24 h [49].

A study by Di Biase et al. compared lesion durability and collateral damage between focal unipolar/biphasic pulsed field ablation and RF ablation in swine, as limited data exist on point-by-point PFA. Eighteen swine were randomized into low-dose PFA, high-dose PFA, and RF groups. Using a multimodality generator, RF served as the control group. A contact force-sensing catheter guided focal PFA/RF delivery at predefined atrial sites. Postprocedural and 28-day remapping confirmed successful ablation with persistent PVI and durable lesions at non-pulmonary vein sites. Lesion sizes were consistent, achieving transmurality in 95% of sites. PFA animals showed mature scar formation on histology without necrosis or inflammation, and no collateral damage was observed. This preclinical study suggests that focal unipolar/biphasic PFA guided by contact force values results in durable lesions, mature scar formation, and no collateral injury, indicating potential for further investigation in AF treatment [50].

Using thermal ablation for PVI can lead to severe complications like esophageal damage, including atrio-esophageal fistulas. However, in a study involving 84 New Zealand rabbits where non-thermal irreversible electroporation was applied to their esophagi, there were no reports of lumen stenosis, epithelial issues, ulcers, or fistulas 16 weeks after the procedure [51]. These results are confirmed in another study that demonstrated that esophageal cells could regenerate through self-replication within 4 weeks with complete anatomical repair achieved through structural remodeling and no occurrence of lumen stenosis, ulcers, or fistulas [52].

Another study conducted by Buist et al. [53] investigated the feasibility of irreversible electroporation (IRE) ablation in the coronary sinus (CS) using a porcine model for persistent AF. Previous research highlighted the CS as a crucial target, but RF ablation in the CS has raised concerns about complications like coronary vessel damage. Animal data suggested IRE ablation as a potentially safe alternative near coronary arteries. Ablation and pacing were performed in the CS of six pigs using a modified catheter, with pacing maneuvers assessing atrial capture thresholds. Twenty-seven IRE applications were executed without complications during the 3-week survival period. Postablation, 100% isolation was achieved, and pacing thresholds at the 3-week follow-up were significantly higher. Histological analysis revealed transmural ablation lesions in the CS’s muscular sleeves. In conclusion, IRE ablation using a multielectrode catheter proved feasible in the CS, suggesting a promising direction for safer ablation modalities in proximity to coronary arteries [50].

A systematic review of 16 animal studies evaluated irreversible electroporation’s efficacy and safety in preclinical settings. Irreversible electroporation was applied to various areas including the ventricular myocardium, atrial tissue/pulmonary veins, coronary arteries, esophagus, phrenic nerve, and cardiac ganglia, totaling 320 ablations. Histologically, larger and more transmural lesions correlated with higher energy, pulse amplitude, longer pulses, or increased pulse numbers, particularly in the ventricular myocardium and atrial tissue. Ablation of ganglia was successful in 83% of cases, while vessels (coronary arteries) showed no signs of ulceration or adverse reactions. The esophagi also displayed no documented adverse effects [54].

Given the success of this new technology on animals, various catheters have been developed and tested over time.

A study introduced an 8F lattice-tip catheter (Sphere-9™, Affera Inc., Newton, MA, USA) developed for single-shot PV and PW using PFA. The catheter, expandable up to 34 mm, applied PFA through six elements using a biphasic waveform in swine models targeting the superior vena cava (SVC) and right superior pulmonary vein (RSPV) for isolation. Animals were observed for short-term (12–24 h) and long-term (3 weeks) safety and efficacy. PFA successfully isolated SVC and RSPV without significant regression or expansion after the survival period. Only one instance of sinus node arrest occurred, while phrenic nerve function remained unaffected, and minimal bubble formation was observed. The study indicates that this expandable lattice catheter achieved rapid and enduring PVI in preclinical testing [55].

## 4. Safety Profile of Pulsed Field Ablation

In the IMPULSE study, primary adverse events were noted in 2.5% of patients, including two cases of pericardial effusions or tamponade, one hematoma, and one transient ischemic attack (TIA). The 1-year Kaplan–Meier estimates for freedom from any atrial arrhythmia were 78.5 ± 3.8% for the entire cohort and 84.5 ± 5.4% for the cohort using the optimized biphasic energy PFA waveform [56,57].

In the PULSED AF trial, involving 300 patients with paroxysmal or persistent AF refractory to AADs, the primary safety endpoint (a composite of acute procedural failure, arrhythmia recurrence, or antiarrhythmic escalation over 12 months, excluding a 3-month blanking period to allow recovery from the procedure) occurred in only 0.7% of patients in both cohorts. The trial suggests that PFA, utilizing irreversible electroporation, provides effectiveness comparable to established ablation technologies for treating AF, with a notably low rate of primary safety adverse events [58].

In one of the main retrospective surveys on PFA, the MANIFEST-PF trial included 24 clinical centers utilizing the pentaspline PFA catheter following regulatory approval. The study involved 1758 patients with an average of 73 treated per center (range 7–291). The patients had a mean age of 61.6 years (range 19–92), with 34% females, 94% undergoing their first ablation, and 58% and 35% having paroxysmal and persistent AF, respectively. The majority of procedures (82.1%) utilized deep sedation without intubation, and 15.1% of patients were discharged on the same day. No esophageal complications or persistent phrenic nerve injuries were reported beyond hospital discharge. Major complications occurred at a rate of 1.6%, including pericardial tamponade (0.97%) and stroke (0.4%), with one stroke leading to death (0.06%). Minor complications occurred in 3.9% of cases, primarily vascular (3.3%), but also featuring transient phrenic nerve paresis (0.46%) and TIA (0.11%). Acute major adverse events occurred in 1.9% of patients [59]. Rare complications, each at a rate of 0.06%, included coronary artery spasm, a phenomenon that can be attenuated by nitroglycerin, administered either post hoc to treat spasm or as prophylaxis [60], hemoptysis, and a persistent dry cough lasting for 6 weeks. In summary, this extensive evaluation of an unselected patient cohort revealed the efficacy of PFA in PVI, demonstrating a safety profile consistent with preferential tissue ablation. However, the occurrence of “generic” catheter complications such as tamponade and stroke emphasizes the imperative for further enhancements in procedural safety [57,61].

Another systematic review from Shtembari et al. evaluates the safety and efficacy of PFA compared to thermal ablation for treating AF. Six studies (including the MANIFEST-PF survey) involving 1897 patients undergoing PFA were analyzed. Successful PVI was achieved in 100% of cases, with rare major complications such as pericardial tamponade, vascular issues requiring surgery, and stroke. Atrial arrhythmia recurrence was lower in the PFA group (11%) compared to the thermal ablation group (39%). The findings suggest that PFA demonstrates a high success rate in PVI with few major adverse events, and it reduces atrial arrhythmia recurrence compared to thermal ablation [62]. Vascular access site hemorrhage (*n* = 1/38) and cardiac tamponade/perforation (*n* = 1/25) were the serious adverse events reported by Verma et al. [63] and Reddy et al. [64], respectively. In another study by Reddy VY et al. [65], both major and minor complications were observed, which included major vascular complications requiring surgical repair (*n* = 1/76, 1.3%) and minor complications like esophageal erythema (*n* = 2/60, 3.3%) and minor vascular complications (*n* = 4/76, 5.3%). In the study by Nakatani Y. et al., a minor complication was groin hematoma in both PFA (*n* = 1, 6%) and thermal groups (*n* = 2, 9%), which resolved on conservative management [66].

Despite the well-established safety profile of PFA, several aspects related to this innovative technique still require improvement. For instance, in some comparative studies between PFA and RFA regarding the incidence and severity of left circumflex arterial vasospasm during adjacent ablation along the mitral isthmus (MI), vasospasm was found to be frequent with PFA and not with RFA, but it was typically subclinical [67,68]. Another study by Kirstein B et al. investigated intraluminal esophageal temperature changes during PFA for AF. In a cohort of consecutive patients, a median temperature increase of 0.8 ± 0.6 °C was observed, with 23% experiencing a rise of ≥1 °C. The highest recorded temperature was 40.3 °C. Despite these changes, no symptomatic esophageal injuries or atrio-esophageal fistulas were reported in follow-up. Further research is warranted to fully understand the clinical implications of these temperature variations during PFA [69]. 

Moreover, after two instances of acute kidney injury (AKI) attributed to hemolysis following PFA procedures that occurred in May and June 2023, the group of Sandrine Venier et al. conducted an evaluation of hemolysis in a consecutive patient cohort, in which 68 consecutive patients (mean age 64.3 ± 10.5 years) undergoing AF ablation with PFA were included. Blood samples were collected from all patients the day after the procedure to assess hemolysis indicators. The pentaspline PFA catheter was used with a median total application number of 64 (54; 76). Nineteen patients (28%) exhibited significantly reduced haptoglobin levels (<0.04 g/L). A notable inverse correlation was identified between plasma haptoglobin levels and the total number of applications. Two groups were compared: the hemolysis+ group (haptoglobin < 0.04 g/L) vs. the hemolysis− group. The hemolysis+ group showed a significantly higher total number of applications compared to the hemolysis− group, with respective values of 75 (62; 127) vs. 62 (54; 71) (*p* = 0.011). The use of more than 70 applications appears to have improved sensitivity and specificity for predicting hemolysis. Intravascular hemolysis can occur after specific PFA procedures, with AKI being a rare phenomenon. However, caution is advised regarding the number of applications to prevent severe hemolysis [70].

A study of Mohanty, Sanghamitra et al. focused on the development of pulmonary hypertension (PH) following reduced LA compliance after AF ablation. The objective was to compare the risk of worsening baseline PH between non-PAF patients undergoing PFA and standard RF ablation. In this multicenter study, 28 non-PAF patients with PH underwent PFA-based ablation after more than one failed RFA and were compared to a control group of 28 AF patients with PH scheduled for repeat RFA. After the ablation, there was no detected worsening of mean pulmonary artery pressure (mPAP) in the PFA group, suggesting that this technique does not exacerbate preexisting PH compared to repeat RFA [71].

## 5. Pulsed Field Ablation: Clinical Studies

The first clinical use of PFA for AF ablation was described in 2018 by the group of Reddy et al. [72] (see Table 1 for the list of the cited studies). Their case series includes 15 patients suffering from PAF treated by endocardial ablation with PVI and 7 patients who, undergoing cardiac surgery for another reason and suffering from AF, underwent an epicardial ablation of the AF with electrical isolation of the PV and of the posterior wall (PW); to date, it is the only clinical experience of epicardial ablation using PFA. The ablation showed intraprocedural success in the electrical isolation of the PV of 100% in the first group and 85% in the second group (isolation of the PV and PW); the ablation times were very short (average procedure time in the first group: 67 ± 10 min; average ablation time in the second group: 50 ± 19 min) and no complications related to the procedure were reported, particularly no PV stenosis, stroke or TIA, pericardial tamponade, atrioesophageal fistula, or phrenic nerve injury. However, long-term data on the efficacy and safety of the procedure are lacking.

In 2020, two new case series of patients treated with PFA were published, again by Reddy et al. [60,61]. The first case series [61] included 76 patients, of which 55 were affected by PAF and 21 by permanent AF, treated using a particular type of ablation catheter (lattice-tip ablation catheter, produced by Affera Inc.) with two peculiarities: (1) it is able to release energy both in the form of radiofrequency and PF; (2) it performs point-by-point ablation. These patients underwent two ablation strategies: in 36 patients PF alone was used, while in the remaining 40 both types of energy were used, preferring PF for the posterior cardiac structures in the left atrium. In addition to PVI, further ablations were performed, like cavotricuspid isthmus (CTI) or, in patients with persistent AF, posterior MI, left atrium roof, per operator discretion. Furthermore, most patients underwent esophagogastroduodenoscopy (EGDS) and brain MRI in the days following the ablation to exclude silent esophageal or brain lesions. Electrical isolation of the PVs was achieved in 100% of cases and the success rate of further linear lesions was also approximately 100%; only one patient experienced a significant adverse event, a left groin hematoma that required surgical correction. EGDS excluded significant damage to the esophagus in patients undergoing this examination, while brain MRI detected a low percentage of asymptomatic cerebral ischemic phenomena (9.8%) of patients undergoing MRI, in line with previous reported incidence ranges (using different energy modalities) of new cerebral ischemic events between 2% and 40% [73,74]. Even in this case study, long-term data are missing.

The second case series [60] included 25 patients suffering from persistent AF: these patients underwent an ablation procedure not limited to isolation of the PV alone but also of the PW of the left atrium and the CTI. Approximately 3 months after the first ablation procedure they were studied again to evaluate the durability of the PFA lesions. Two PFA catheters were used for this procedure (both designed by Farapulse-Boston Scientific Inc. Boston, MA, USA): the Farawave catheter, still the most used catheter today, contains five splines, each containing four electrodes, which can take on two different morphologies: basket-shaped or flower-shaped, used for the isolation of the PV and the PW; a deflectable focal Faraflex catheter with four short splines, each containing four electrodes. It adopts configurations ranging from oblong to spherical, mainly used for CTI ablation. The first procedure showed 100% acute success for isolation of the PV, PW, and the CTI line block, with an average duration of 125 min. Of the 25 patients, 22 underwent the remapping procedure after 3 months which highlighted the persistence of electrical isolation in 96% of the PVs, in 100% of the PW, and 75% of the CTI block line. The only adverse event that occurred was a pericardial effusion, which did not lead to cardiac tamponade and which occurred during remapping with an RF catheter and therefore was not related to the PFA. Following the procedure, 21 patients underwent EGDS and 14 underwent CT; no damage to the esophagus nor PV stenosis were highlighted.

The first data on the long-term efficacy of PFA were published in 2021 by Reddy et al. [56]. This study examined a group of subjects enrolled in three different non-randomized, prospective studies: IMPULSE, Pefcat, and Pefcat II. Totally, 121 patients suffering from PAF underwent PFA, using the already described single-shot PFA Farawave catheter. In only three patients, coming from the Pefcat II study, the focal PFA Faraflex catheter was also used for the ablation of the CTI. Different PFA ablation waveform and delivery protocols were employed depending on the studies. Approximately 3 months later, 110 patients underwent new remapping to evaluate the effectiveness of PFA; clinical follow-up was extended to 1 year, during which onset of atrial arrhythmias, including AF, atrial flutter (AFL), and other forms of atrial tachycardia (AT) was assessed. All PVs were isolated during the ablation procedure, with an average duration of 96 min, including a 20 min wait to confirm vein isolation. At 3 months, 84.8% of PVs were isolated. In patients who received optimized PFA, the PV isolation rate was also higher (96%). The line block on CTI was confirmed in two out of three patients. This difference in procedural success was also confirmed by the Kaplan–Meier curves which at 1 year showed a higher percentage of freedom from atrial tachyarrhythmias (AF, AFL, AT) in patients undergoing optimized PFA compared to all patients, 84.5 ± 5.4% vs. 78.5 ± 3.8%, respectively. The data on the safety of PFA were also very positive: a total of four patients experienced adverse events (one cardiac tamponade, one pericardial effusion, one vascular hematoma, and one acute ischemic attack). At CT/MRI follow-up, no patient developed pulmonary stenosis; no esophageal lesion was visualized by EGDS, nor by MRI, in which it was appreciated that the LGE remained confined to the left atrium following ablation. Of the 18 patients who underwent brain MRI, two acute lesions were found: of these, one had caused no symptoms, while the other was responsible for the TIA previously described.

These first clinical studies confirmed data on the acute efficacy of this new form of energy for the ablation of AF and its selectivity on myocardial tissue, without damaging the surrounding structures; nevertheless, the limitation of the sample and the selectivity of the centers that used PFA did not guarantee the feasibility and safety of PFA in clinical practice. In this regard, after regulatory approval and commercialization of the pentaspline PFA Farawave catheter (Farapulse-Boston Scientific Inc.), in 2021, the first data have emerged on its use in routine practice [66].

The largest case series was described in MANIFEST-PF, a retrospective survey that included all European centers that performed PFA in clinical practice with the Farawave catheter [61]. In the 24 European centers involved, with an average number of 3.8 operators per center, 1758 patients were enrolled and, of these, approximately 60% were affected by paroxysmal AF and 93.5% underwent AF ablation for the first time. In addition to isolation of the PVs, a minority of patients underwent isolation of the roof or PW of the LA or ablation of the posterior perimitral isthmus. Only in some patients was pre- and postablation electroanatomical mapping of the left atrium performed and no preventive measures to protect the esophagus were used. The survey showed that the electrical insulation of the PVs was completed in 99.9% with an average duration of 65 min per procedure; no data regarding additional lesions in the left atrium were mentioned. Regarding safety data, 1.86% of patients experienced major complications, of which 0.97% suffered cardiac tamponade and 0.36% suffered stroke. Among these, there were no cases of esophageal damage pulmonary stenosis or permanent phrenic nerve damage (only transient phrenic nerve paresis in eight patients) and one patient died as a result of stroke (0.06%). The rate of minor complications, however, was 3.86%, almost all due to vascular complications and, to a lesser extent, TIA. These safety data confirmed the selectivity of PFA on cardiac tissue and also showed an excellent safety profile of this new method in clinical practice, as the complications that occurred do not appear to be attributable to the use of PFA. However, a case of coronary spasm with associated ST-segment elevation has been described during the use of PFA at the level of the posterior MI, which then regressed with nitroglycerin. Follow-up data on efficacy are lacking.

Subsequently, in 2023, a new multicenter European registry, called EU-PORIA, showed the real-world results of AF ablation using PFA in a population composed of 1233 patients, of which 60% were affected by PAF [75]. This study highlighted an acute efficacy of 99.6% in PVI, with an average duration of the procedure of 58 min. Also in this study, only a minority of patients underwent electroanatomical mapping and in 14% of cases additional lesions were operated on in addition to PVI. The complication rate related to the procedure was 3.6%, for a total of 45 adverse events: of these, 21 were considered major events, divided between 14 pericardial effusions and 7 acute cerebral events. During follow-up, 149 patients underwent a repeated ablation procedure due to the occurrence of relapses of supraventricular arrhythmias (99 for AF and 50 for AT): upon remapping, the isolation of the PVs persisted in 72% of the veins and in the 36% of patients. At 12 months, the Kaplan–Meier estimate of AF/AT-free survival was 74% for the total cohort, 80% in patients with PAF at the procedure, and 66–67% in patients with persistent AF.

The first randomized, single-blind clinical trial in which PFA was compared to thermal ablation in the transcatheter treatment of PAF was the ADVENT trial, published by Reddy et al. [76]. The authors enrolled 607 subjects suffering from PAF resistant to drug therapy and randomly assigned them to PFA or thermal ablation, either RF or CB. The efficacy and safety endpoints used to demonstrate non-inferiority of PFA were: acute success of PVI, documented atrial tachyarrhythmia lasting at least 30 s, the use of Class I or III AADs or cardioversion after the 3-month blanking period, the use of amiodarone at any time or repeat ablation regarding efficacy. Serious adverse events related to the procedure and shrinkage in the aggregate pulmonary vein cross-sectional area between baseline and 3 months were safety endpoints. 

PFA proved to be non-inferior to thermal ablation both in terms of intraprocedural efficacy, 99.6% for PFA vs. 99.8% for thermal ablation, and for one-year efficacy, evaluated with the endpoints described above, with a treatment success rate of 73.3% vs. 71.3% for the other two methods. Also, with regard to safety, the non-inferiority of PFA was demonstrated (six adverse events in patients undergoing PFA vs. four in patients undergoing thermal ablation; this difference was not statistically significant). Narrowing of the PV ostia was analyzed in the majority of patients: PFA was superior in minimizing this complication. In addition, the ablation procedure was faster with the use of PFA (106 ± 29 min with PFA vs. 123 ± 42 min with thermal ablation).

Another study, published by Urbanek et al. [77], compared PFA with CB ablation in a cohort of 400 patients in total, with approximately 60% affected by PAF, subjected to PVI with one of the two strategies (200 subjects for each method). The acute success rate was approximately 100% for both energy sources, with a lower average duration of the procedure in the PFA group (34.5 min vs. 50 min). Even in the long term the two methods were similar, with overall freedom from AF after 1 year of 78.1% in the CB group and 74.5% in the PFA group, percentages that remained similar when comparing the types of AF (PAF: 83.1% in CB and 80.3% in PFA; persistent AF: 71% in CB and 66.8% in PFA). In patients in whom the procedure was repeated due to recurrence of arrhythmia, 16 patients in the CB group and 26 in the PFA group, the veins were persistently isolated in more than 80% of cases in both groups (82.8% in CB group, 85.6% in the other group). The complication rate was higher in the group subjected to CB compared to PFA (6.5% vs. 3%, respectively), a difference mainly due to injury of the surrounding tissues, in particular there were three persistent phrenic nerve palsies and one thermal esophageal injury following the CB.

A recent analysis, written by Della Rocca et al., compared the effectiveness of PFA in PVI with the other two ablation technologies, RF and CB [78]. In four different centers, based on clinical and demographic characteristics to minimize possible interferences, 860 subjects suffering from PAF were selected and subjected to one of the three ablation technologies, with a ratio of 1:2:2 for PFA, RF, and CB, respectively. This study highlighted 100% effectiveness in electrical insulation of PV of all three technologies, a shorter average duration for PFA (average procedure duration of 52 ± 14, 64 ± 22, 85 ± 25, respectively, for PFA, CB, and RF), and a statistically significant difference for the occurrence of minor complications with the use of PFA, while major complications were equally infrequent in the three groups. At one year of follow-up, event-free survival is similar in the three groups, all above 70%, while PFA showed a trend towards statistical significance for greater freedom from AF recurrences (PFA: 85.5% vs. CRYO: 78.5% vs. RF: 77.4%; log-rank *p*-value = 0.09). Furthermore, in patients who underwent a repeated ablation procedure, approximately 20%, the rate of electrical reconnection of the PVs was higher for the two thermal ablation technologies compared to PFA (19.1%, 27.5%, and 34.8%, respectively, in the groups subjected to PFA, CB, and RF).

Davong et al. investigated the feasibility and safety of MI ablation with the use of PFA in patients with persistent AF [79]. They performed MI ablation, on top on PVI and PW isolation, in 45 patients through applications in a flower configuration of the Farawave catheter, extending from the mitral annulus to the left inferior pulmonary vein. PV, PW, and MI were successfully ablated in all cases, on average with 85 ± 23 deliveries, of which 31 ± 17 completed the MI block line. During the follow-up of approximately 100 days, nine patients (20%) experienced recurrences: reconnection of the MI was found in all four patients who underwent a new ablation procedure. A low frequency of adverse events was reported in this study, 6.6% (only three events): one air embolism and two cases of coronary spasm of the circumflex artery, which occurred during MI ablation and promptly regressed after nitroglycerin infusion. This complication had already been described in the MANIFEST-PF survey [59].

Venier et al. described two episodes of AKI in two patients subjected to more than 100 applications of PFA for the transcatheter treatment of forms of persistent AF, extending the ablation to the PW and MI as well as to the PVs [70]. The mechanism underlying renal damage consisted of massive hemolysis responsible for hemoglobinuria and necrosis of the renal tubules. The authors subsequently evaluated the predisposing factors for the occurrence of hemolysis after PFA procedures, identifying a high number of pulsed field applications as the only risk factor, outlining the value of 70 deliveries as the ideal cut-off to avoid this complication.

In the last year, new clinical studies have also been published on other PFA catheters in addition to Farawave [57,80]. The PULSED AF Pivotal trial, non-randomized clinical study, evaluated the efficacy and safety of the Medtronic PFA catheter in PVI [58], initially evaluated in the PULSED AF Pilot Trial which included a limited number of subjects. This consists of a circular catheter containing nine electrodes that deliver biphasic, bipolar energy. This study included 300 patients, exactly half of whom suffered from PAF and the other half with persistent AF, and performed careful follow-up to identify the recurrence of arrhythmias. Intraprocedural efficacy was 100%, with a device left atrial dwell mean time of 65 ± 29 min for paroxysmal forms and 70 ± 31 for permanent forms. Only two serious complications due to the procedure were documented, a pericardial effusion and a cerebrovascular event, while no damage to the surrounding structures was highlighted. The efficacy of the procedure at 12 months, considering a complex endpoint including acute success of the procedure, recurrence of arrhythmias, increased or new use of AADs, and repeat ablation or cardioversion, was 67% in PAF and 55% in persistent cases, also obtaining a significant increase in the quality of life.

The inspIRE trial evaluated the performance of the Biosense catheter, the first catheter that works in combination with a mapping system, in a cohort of 226 patients suffering from PAF and subjected to PVI [80]. It is a decapolar irrigated circular loop catheter, similar to the Medtronic catheter. The trial was divided into two phases: the first which certified the safety of the procedure in 40 patients and the second which included the remaining patients. The results of the study documented 100% acute efficacy and no adverse events; considering only the second phase of the study, the procedure lasted on average 70 ± 27 min and at 12 months 70.9% of patients were free from recurrence.

A new multicenter clinical trial evaluated the RF/PF lattice-tip point-by-point ablation catheter, produced by Affera Inc. [81]. This trial enrolled 178 patients, the majority suffering from persistent AF, and as in previous clinical experience with this catheter [65], patients underwent PVI and of further lesions along the left atrium, if necessary, using PFA exclusively for applications at the level of the posterior structures of the LA and one of the two technologies for the anterior structures. During follow-up, additional tests, such as EGDS, brain MRI, and lung CT, were often used to identify possible adverse events. The effectiveness of this catheter for PVI and additional block lines was 100%, with an average procedure duration of 99 ± 34 min; at approximately 3 months, 69% of patients underwent remapping, confirming isolation in 75% of the pulmonary veins, a percentage that reached 97% when the PFA optimized waveform was used. At 12 months, the percentage of patients free of recurrence was 78 ± 5% and 77 ± 4% for the paroxysmal and persistent AF subgroups, respectively. Five patients experienced adverse events related to the procedure, of which only one was attributable to the device. During instrumental examinations, the following were reported: three cases of minimal thermal damage to the mucosa of the esophagus, all asymptomatic and appearing in patients in whom RF was used. New silent brain events occurred in 7.9% of patients undergoing brain MRI. There were no cases of PV. These results therefore confirmed the safety of this new PFA catheter.

Another new system allows for an integration of PFA with preexisting mapping programs. A connector, which links normal RF catheters with a pulsed electric field generator, allows the use of this new form of energy, without giving up the aid of electroanatomical mapping and contact force-sensing. This system is called the Centauri system and was evaluated in the ECLIPSE AF study [82] which analyzed the efficacy and safety of this system in a cohort of 82 patients suffering from paroxysmal or persistent AF, approximately equally divided. Patients underwent PVI and after 90 days a new procedure to confirm PVI. After a first phase of the study in which the most effective ablation strategy was evaluated, in the second phase patients underwent an optimized PFA. The study showed an intraprocedural efficacy of 100% in PVI, with an average procedure duration of 140 ± 42 min. Electrical isolation was durable in 89% of PVs in patients who received an optimized PFA. Four patients experienced serious adverse events, while two patients developed pericardial effusion and cardiac tamponade. No significant damage to the structures surrounding the heart was demonstrated and on brain MRI new silent brain events were identified in 11.4% of cases undergoing MRI (35 patients).

Nakatani et al. [66] analyzed the effects of PFA on the structural and functional characteristics of the LA compared to those produced by thermal ablation based on the information collected by cardiac MRI. In a small cohort of patients undergoing AF ablation, some treated with PFA and others with RF/CB, three different cardiac MRIs were performed, one before ablation, one within 3 h of ablation, and, finally, one after 3 months. In both groups, they observed an acute increase in LGE at the LA level, greater in the group subjected to PFA and without signs of signal inhomogeneity, suggestive of intramural hemorrhage and microvascular damage, present instead in the group subjected to thermal ablation. At 3 months, the portion of LGE decreased significantly in the PFA group, which did not happen in the other group, without this affecting the durability of the lesions: in fact, all patients in the PFA group underwent a remapping which confirmed the electrical insulation of the veins. These results were also associated with a recovery of the atrial strain and therefore of the functionality of the LA only in subjects undergoing PFA. These data confirm the high selectivity of PFA on atrial myocytes and a lower inflammatory and reparative response in this new type of ablation.

With a similar technique, based on the acquisition of three MRIs per patient, Cochet et al. [35] investigated the effects of PFA on the structures surrounding the LA. In a limited number of patients, they did not observe any acute lesions on the esophagus in patients who underwent PFA, while new lesions were observed in 33% of patients who underwent thermal ablation. On the contrary, new lesions were documented in both groups on the descending aorta, in 33% and 43% of patients undergoing PFA and thermal ablation, respectively. These lesions did not cause any clinical effects and regressed after three months, confirming the safety and tissue selectivity of PFA.

The selectivity of PFA on atrial myocardiocytes, sparing the nervous structures, was investigated by Lemoine et al. [83], who measured the change in tissue-specific biomarkers and resting heart rate following the ablation procedure in 91 patients, treated via either PFA or CB. They highlighted that troponin I increased much more after PFA than after CB, unlike the neuronal biomarker S100B protein, which increased more after CB. Furthermore, an increase in baseline HR occurred only after CB. These results confirmed the cardiomyocyte specificity of PFA, as had been suggested by animal models.

Tohoku et al. first evaluated the characteristics of relapsing tachyarrhythmias post-PVI with the pentaspline PFA catheter [84]. In 25 patients undergoing a second ablation procedure, the most frequent arrhythmic recurrence was atrial tachycardia due to macro-reentry. Confirming this, they found an overall low rate of PV reconnection (9.1%). The critical isthmus of the macro-reentry in most cases resided in the PW which in these patients had a large percentage of surface included in the area electrically isolated from the previous ablation; this determined a narrow zone of viable myocardium in between the previous PVI lesions. These findings suggest individualized lesion planning and lesion reduction in some patients to minimize the risk of tachyarrhythmia recurrence. In the same study, the authors observed a higher rate of reconnection in the upper left PV (16%) and that the increased diameter of the PV and the use of the 35 mm catheter were associated with an increased risk of reconnection.

Other studies described preferential regions of PV reconnection and the results were not unambiguous. In the 2021 case series by Gunawardene et al. [85], 20 patients underwent high-density electroanatomical mapping at the end of the PFA procedure. They located all PV reconnections in the anterior regions of the superior PV, for a total of 6.25% of reconnected veins.

Ruwald et al. [86], in 26 patients with recurrence of atrial tachyarrhythmia post-PFA undergoing a new ablation procedure, observed a PV reconnection rate of 31% and greater recurrence in the right PV; in particular, the right PV carina was the region with the highest rate of PV reconnection [86].

In the case series of Magni et al. [87], including 14 patients undergoing a redo AF ablation procedure, the right inferior PV was the vein most electrically reconnected to the LA (in patients with at least one reconnected vein, the right inferior PV was involved in 50% of cases). Electrical isolation of the veins persisted in 64.2% of PV.

In the work of Kueffer et al. [88], in 29 patients subjected to a redo of AF ablation, 63% of the PV remained isolated and, as in the two previous studies, reconnections occurred more in the right PV.

Instead, Della Rocca et al., in their previously mentioned analysis [78], observed a greater rate of reconnection in the left superior PV (27%).

A recent work by Kueffer et al. [88] showed the results of a large series of 144 patients undergoing a redo procedure, coming from the EU-PORIA registry, already cited and analyzed previously [75]. Seventy-one percent of PV were isolated and there was no significant difference compared to the four PV. Instead, analyzing the specific sites of the reconnections, these occurred mostly in the anterior portions of the superior PV and in the inferior portion of the right inferior PV, 40% and 21% of the total reconnections, respectively. Furthermore, they saw that older age, less atrium dilatation, and operator’s prior experience with cryoablation were predictors of durable PVI.

## 6. Pulsed Field Ablation: Catheters and Ablation Procedure

As previously reported, the only PFA ablation catheter with regulatory approval for use in clinical practice is the Farawave catheter, manufactured by Farapulse-Boston Scientific Inc.

This 12F over-the-wire catheter is composed of five splines, each contain four electrodes, and can be deployed in flower petal or basket configuration. There are two possible catheter sizes, based on the maximum diameter of the distal portion of the catheter when fully deployed in the flower configuration: 31 or 35 mm. All 20 electrodes contained on the splines release the pulsed fields; in contrast, only the third electrode of each spline is capable of recording electrograms [55,57,89]. The particular design of the catheter allows for a single-shot ablation.

In addition to the catheter, a 13-F steerable sheath with clear shaft, called Faradrive, and a custom generator, Farastar, which delivers ultrafast, high-voltage electrical pulses across multiple channels, leading to selective tissue electroporation, are required. The magnitude of the PF voltage could vary between 1.8 and 2.0 kV, but typically 2.0 kV was used [66].

The ablation procedure is performed under sedation or general anesthesia, maintaining activated clotting times at a minimum of 300 s using intravenous heparin. After performing the transseptal puncture, the Farawave catheter is advanced into the left atrium via the 13-F sheath and, once the guide has been positioned in each PV target, the catheter is positioned at the ostium of the respective vein, under fluoroscopic guidance or, more rarely, via intracardiac echo. Then, at least eight deliveries per vein are performed: four deliveries in the flower shape and four in the basket shape, and after every two deliveries the catheter is rotated by approximately 36 degrees. Isolation of the veins is confirmed by entry and exit blocking. In addition, to reduce the risk of recurrence, especially in persistent AF, further applications can be made to obtain the isolation of the PW and the block line of the MI, using the flower shape of the catheter. Normally, no preventative measures are used to protect the esophagus during the procedure.

The Medtronic PSA system uses a 9-F over-the-wire catheter, but circular, composed of nine electrodes and which reaches a diameter of 25 mm [65,74]. The catheter is capable of both pacing and sensing and, due to a gap between the first electrode and the ninth, must be rotated to obtain complete electrical isolation of the veins. The system releases biphasic, bipolar pulse trains, each lasting 100 to 200 ms at 1400 to 1500 V measured from baseline to peak.

The Biosense catheter is also circular and made up of 10 electrodes, all of which are capable of recording, stimulating, and delivering PFA [82]. The catheter, steerable and irrigated, is 8.5-F and the distal loop can be expanded or retracted to better adapt to the anatomy of the PV. It delivers PFA in a bipolar configuration with an energy of 1800 V; the entire PFA Biosense system is compatible with the electroanatomical mapping system. The last two catheters belong to the single-shot ablation technology.

The two PFA systems that allow point-by-point ablation are the Centauri and Affera systems. The Centauri system, as already described, uses traditional RF scaling catheters which, thanks to a connector, can release pulsed electric fields, maintaining the possibility of carrying out electroanatomic mapping [80]. The system designed by Affera Inc., however, is the only catheter capable of releasing both RF and pulsed electric fields [65,80]. It includes two RF and PEF generators, a mapping system, and a lattice-tip ablation catheter (“Sphere-9”); the latter is a 7.5-F bidirectional deflectable catheter which, in its distal portion, has an expandable 9 mm diameter nitinol lattice electrode, containing nine mini electrodes (0.7 mm diameter each) on the spherical surface and a neutral non-contact electrode inside. Two additional ring electrodes are located proximally on the shaft. Both energy forms are released from the expanded form of the lattice tip. The mini electrodes, equipped with thermal sensors, control the temperature reached during the delivery of RF, while they have no role during the release of the PF. The system releases a biphasic PF waveform, composed of a train of microsecond-scale pulses delivered over 3 to 5 s.

## 7. Discussion

PFA, although its use in AF ablation is rather recent, has already accumulated large scientific evidence on its safety and effectiveness. The first published clinical studies highlighted high efficacy of PFA in the ablation of AF, both in paroxysmal and persistent forms, obtaining intraprocedural success rates close to 100% in PVI and also very high for other lesions in the LA [56,60,61,68]. These results were obtained with few or no complications. In particular, the lesions operated on by PFA did not involve the structures surrounding the atrial tissue, unlike thermal energy ablation [8,90]. Following the commercialization of the PFA Farawave catheter, the MANIFEST-PF survey and the EU-PORIA European registry showed the positive results of this method in the real world [61,75]. In addition to almost total intraprocedural success, the average duration of the procedures was very short, 58 min in MANIFEST-PF and 65 min in EU-PORIA, much shorter than the duration of AF ablation with RF or CB indicated in the large trials (average duration between 121 and 174 min) [22,23,24]; this translates into greater accessibility of ablation in daily clinical activity, given the high prevalence of AF and the growing demand for ablation. The safety data were also very exciting: complication rates of 5.72% for MANIFEST-PF and 3.6% for EU-PORIA, in line with, if not lower than, what was reported for ablation via thermal energy, confirming an excellent safety profile of this energy source [8,91]. PFA essentially spared the tissues surrounding the heart, causing no significant damage to the nervous structures, esophagus, and pulmonary veins. The latter is a rare but feared complication in RF and CB ablation. As discussed previously, only exceptionally did PFA have effects on surrounding tissues: cases of transient paresis to the phrenic nerve were described in MANIFEST-PF (0.46% of cases) [59]; Kirstein et al. observed an increase in esophageal temperature during PFA which had no clinical repercussions in follow-up [69]; three cases of coronary spasm, one of hemoptysis, and one of persistent dry cough were described [57,79]. 

In addition, Venier S et al. described two cases of acute kidney injury due to hemolysis following massive use of PFA, without, however, causing persistent damage to renal function. The authors identified a correlation between the number of PFA applications and the decrease in haptoglobin levels [70]. Two studies highlighted the lower risk of adverse effects on the pulmonary venous circulation of PFA compared to thermal energy ablation: the group of Sanghamitra [71] did not observe any effect on the average pressures of the pulmonary circulation, while in the ADVENT trial [87], PFA was associated with less narrowing of the caliber of the PVs.

From what has been reported, a very encouraging safety profile of PFA emerges. Nonetheless, further research and long-term data will allow us to better evaluate and understand the adverse effects of this new form of energy and to implement all measures to prevent them.

Subsequently, three recent studies, including a randomized trial, compared PFA with RF ablation or CB. All three studies documented a similar effectiveness of PFA in decreasing the recurrence rates of AF/AT/AFL at 1 year, with percentages ranging from 73–79.5%. Similar rates were also described in the EU-PORIA registry, which identified a 1-year event-free rate of 74% [75]. These results are comparable to those reported in previous trials with thermal energy ablation [22,23,24]. Furthermore, PFA was associated with shorter procedural times compared to the other two technologies and was also not inferior in terms of safety endpoints.

In some of the previously cited studies, a minority of patients were subjected to injuries other than PV with PFA [56,60,61]; these may be necessary to study the success rate especially in cases of persistent AF. But the first work that specifically analyzed the validity of the electrical isolation of the PW and especially of the MI with the PFA was published by Davong et al. [79]. They observed 100% intraprocedural success with a low complication rate (6.6%). This result is very encouraging considering that in the literature the success rate of MI block with other energy sources varies from 31% to 92% [92,93] and that different strategies have been used to complete the block, like the alcoholization of Marshall’s vein. Further research and longer follow-ups will be necessary to confirm these initial results.

From the mentioned studies, a low percentage of PV reconnection after PFA emerges, which varies from 6.25% to 37% [77,78,84,85,86,87,88]. Specifically, no sites emerge that are more predisposed to reconnection. Thermal ablation does not show better reconnection rates than PFA, with rates varying from 27% to 54% [94,95,96]. Two of the reported works evaluated the percentages of PV reconnections depending on the energy used for ablation: Urbanek et al. observed similar rates, slightly lower for PFA compared to CB (14.4% and 17.2%, respectively) [77], while in the work of Della Rocca et al. the results are clearly in favor of PFA (19.1% for PFA compared to 27.5% for CB and 34.8% for RF) [78].

Despite the growing diffusion of PFA in electrophysiology laboratories around the world, a standardized protocol for its use that optimizes the risk/benefit ratio of this method has not yet been outlined. Voltage, pulse width, waveform, and polarity are the most important parameters to define for the correct operation of the PFA. Furthermore, greater scientific evidence will be necessary to evaluate and validate the efficacy and safety of PFA on other cardiac structures beyond the PV.

The definition of a standard and optimized protocol, the careful analysis of side effects even in the long term, and the possible peculiarities and advantages of other PFA catheters not yet marketed represent the challenges to be undertaken to make the most of this new and promising form of energy for cardiac ablation.

Nonetheless, all this evidence regarding the intraprocedural success of PFA and the safety of the procedure and its effectiveness, assessed both as the rate of arrhythmia relapses and as the durability of the lesions, increases enthusiasm towards this new form of energy and make it a candidate for an increasingly important role in AF ablation.

## 8. Conclusions

PFA is a new technology that proved to be safe and effective in the treatment of AF. As compared with thermal ablative procedures, electroporation ablation is not associated with worrisome cardiac and non-cardiac complications and represents a promising weapon for electrical PVI and AF ablation.

## Figures and Tables

**Table 1:** List of Studies.

Author, Year and Trial	PFA Catheter	N Patients	Efficacy	% Complication
V.Y. Reddy 2018 [1]	Endocardial and epicardial ablation catheter (Iowa Approach Inc., Iowa City, IA, USA)	22 15 endocardial ablation 7 epicardial abalation	100% PVI by endocardial abatoion 85% PVI by epicardiac ablation	0%
V. Y. Reddy 2020 [2]	RF/PF latex-tip catheter (Affera Inc.)	76	100% PVI	1.3%
V. Y. Reddy 2020 PersAFOne trial [3]	Farawave and Faraflex (Farapulse-Boston Scientific Inc., Boston, MA, USA)	25 persistent AF	100% PVI, PW isolation, and CTI line block 96% persistance of PVI	4%
V. Y. Reddy 2021 Impulse, Pefcat, Pefcat II [6]	Farawave (Farapulse-Boston Scientific Inc.)	121	100% PVI 84.6% persistance of PVI 78.5 ± 3.8% freedom from AF, AFL, AT at 1 year	3.3%
E. Ekanem 2022 MANIFEST-PF [8]	Farawave (Farapulse-Boston Scientific Inc.)	1758	99.6% PVI	1.86% major complications 3.86% minor complications
B. Schimdt 2023 EU-PORIA [9]	Farawave (Farapulse-Boston Scientific Inc.)	1233	99.6% PVI 72% persistance of PVI 74% freedom from AF/AT at 1 year	3.6%
V.Y. Reddy 2023 ADVENT trial [10]	PFA (Farawave) vs. thermal ablation	305 PFA 302 RF/CB	PVI: 99.6% PFA vs. 99.8% RF/CB Freedom from recurrences, increase in antiarrhythmic therapy, or new procedure at 1 year: 73.3% PFA vs. 71.3% RF/CB	1.9% PFA vs. 1.3% RF/CB
L. Urbanek 2023 [11]	PFA (Farawave) vs. CB	200 PFA 200 CB	PVI: 100% in both groups Freedom from AF at 1 year: 74.5% PFA vs. 78.1% CB	3% PFA vs. 6.5% CB
D.G. Della Rocca 2024 [12]	PFA (Farawave) vs. CB vs. RF	172 PFA 344 CB 344 RF	PVI: 100% in all groups Freedom from AF at 1 year: 85.5% PFA vs. 78.5% CB vs. 77.4% RF	3.4% PFA 8.6% CB 5.5% RF
B. Davong 2023 [22]	Farawave (Farapulse-Boston Scientific Inc.)	45 persistent AF	PVI, PW isolation, MI line block: 100%	6.6%
A. Verma 2023 PULSED AF Pivotal trial [13]	Circular catheter (Medtronic, Minneapolis, MN, USA)	300 150 paroxysmal AF 150 persistent AF	100% PVI Freedom from recurrences, increase in antiarrhythmic therapy, or new procedure at 1 year: 67% in paroxysmal AF, 55% in persistent AF	0.67%
M. Duytschaever 2023 inspIRE trial [14]	Circular catheter (Biosense, Irvine, CA, USA)	226	100% PVI 70.9% freedom from recurrences at 1 year	0%
V.Y. Reddy 2023 [15]	RF/PF latex-tip catheter (Affera Inc.)	178	100% PVI 75% persistance of PVI 78% freedom from recurrences at 1 year	2.2%
A. Anić. 2023 ECLIPSE AF [16]	Centauri system	82	100% PVI 89% persistence of PVI after optimized PFA	6%

## Data Availability

Not applicable.

## Abbreviations

Atrial fibrillation	AF
Pulmonary vein	PV
Antiarrhythmic drugs	AADs
Pulsed field ablation	PFA
Transmembrane voltage	TMV
Irreversible electroporation	IRE
High-frequency irreversible electroporation	H-FIRE
Coronary sinus	CS
Superior vena cava	SVC
Transient ischemic attack	TIA
Acute kidney injury	AKI
Pulmonary hypertension	PH
Mean pulmonary artery pressure	mPAP
Posterior wall	PW
Cavotricuspid isthmus	CTI
Atrial flutter	AFL
Atrial tachycardia	AT
Radiofrequency	RF
Cryoballoon ablation	CB
Paroxysmal atrial fibrillation	PAF
Pulmonary vein isolation	PVI
Mitral isthmus	MI
